# Letter from the New Editor

**DOI:** 10.5334/pb.447

**Published:** 2018-04-06

**Authors:** Steve Majerus

**Affiliations:** 1 Université de Liège, BE

**Keywords:** Belgian Association of Psychological Sciences, Psychologica Belgica, Open access, Ubiquity Press

As the new Editor-in-Chief, and together with my new team of associate editors, a group of 15 renowned scientists (https://www.psychologicabelgica.com/about/editorialteam/), I am very honoured and proud to introduce this new volume of Psychologica Belgica. In the line of our predecessors’ work, we aim at consolidating and furthering the international scientific impact of our journal, scientific quality as well as commitment to open science and best research practices being the most important assessment criteria for publication. Psychologica Belgica is an international scientific journal listed by all major publication indexes such as Web of Science, CrossRef, Scopus and Pubmed. We particularly value the submission of research data along with manuscripts and offer several submission formats for research data, either in the form of a specific data or software metapaper or via a dedicated repository such as the Dataverse Network (see https://www.psychologicabelgica.com/about/research-integrity/).

Also, all articles will continue to be published according to our full open access policy at a fair publication fee, in close partnership with our publisher Ubiquity Press (see Van Hiel, 2014). Psychologica Belgica was the first international scientific psychology journal joining Ubiquity Press in 2014, a young company founded by researchers at University College London who believed that science should be available to all the community at no cost and proposing a cost-efficient open access publishing model for authors. After three years of fruitful collaboration, we do not regret our decision: our journal has gained in international visibility as shown by our slowly but constantly increasing impact factor (in 2016, our impact factor was 0.806 and our 5-year impact factor is 1.213). Other well established psychology journals such as the Journal of Cognition, the official journal of the European Society for Cognitive Psychology (ESCOP) have also become convinced by the services provided by Ubiquity Press.

We invite submission of regular empirical articles and theoretical review papers. We also encourage the submission of topics for special issues, which receive a significant amount of attention at both the scientific and media levels. Our last special issue about the Belgian linguistic conflict from a social psychological perspective (Van der Linden & Roets, 2017) has generated nearly 1000 views three months after publication, and has received extensive coverage by the media. We further publish statistical notes that provide a discussion of statistical solutions for complex, non-standard data designs or that introduce novel statistical procedures. This type of submission, launched by our former Editor-in-Chief Etienne Quertemont, can be of enormous help to researchers confronted with statistical questions, and are part of our commitment to good research practices which also include the use of the most appropriate statistical procedures (see Quertemont, 2011a, b).

I also have the pleasure to announce a new publication format, by inviting the submission of original review papers based on recently defended PhD theses. These papers will receive the mention ‘Phd Critical Review’ papers. A huge work goes into the writing of the theoretical part of PhD theses, which present in a comprehensive and critical manner the state of the art of a specific research topic. This type of work can be of tremendous help and save precious time for other researchers planning to do research on a specific research question they are less familiar with. Yet, the rich and critical theoretical background work that characterizes PhD theses is rarely disseminated in the form of a scientific paper. Therefore, Psychologica Belgica encourages researchers who recently obtained their PhD to submit a critical review paper based on the introductory and discussion parts of their PhD thesis. These papers will undergo rigorous peer review like any other type of submission. Like theoretical review papers, these papers should have between 4000 and 10000 words, and should lead to a novel theoretical account and/or to novel research perspectives.

I would also like to remind or inform our Belgian and non-Belgian readers of the fact that Psychologica Belgica is the flagship publication of the Belgian Association of Psychological Sciences (BAPS) whose mission is to support psychological sciences and scientists in both fundamental and applied fields in Belgium (www.baps.be). Psychologica Belgica contributes to positioning BAPS and its members within the international scientific community. I would like to highlight here that regular BAPS members can publish their papers in Psychologica Belgica at no cost (but they need to have been a member of BAPS for at least a 12-month period when submitting their paper in order to be eligible for a publication fee waiver).

In the closing, I am immensely indebted to the excellent work and commitment of the past editorial board headed by Alain Van Hiel. When Alain Van Hiel took office, he had to handle a journal that had just been taken over by our new publisher Ubiquity Press, and he had to manage the transition from our previous paper-only publication format to the new open access publication format. Yet, under Alain’s editorship, the international recognition of Psychologica Belgica has steadily increased. I would like to thank him and his editors (Marc Brysbaert, Laurence Claes, Jean-François Delvenne, Arnaud Destrebecqz, Kristof Dhont, Denis Drieghe, Nicolas Dumay, Valérie Goffaux, Joeri Hofmans, Eva Kemps, Koen Luyckx, Steve Majerus, Koen Ponnet, Jordi Quoidbach, Etienne Quertemont, Bert Reynvoet, Arne Roets, Isabelle Roskam, Bert Timmermans, Bruno Verschuere) for the countless hours they devoted in a selfless manner to their editorial activities. Thanks to their hard work and achievements, it is a real pleasure to act as the new Editor-in-Chief of this journal and I look forward to receiving your contributions from all fields of psychological sciences.
